# Pre-diagnostic serum metabolome and breast cancer risk: a nested case-control study

**DOI:** 10.1186/s13058-025-02102-w

**Published:** 2025-08-27

**Authors:** Ly Trinh, Jaclyn Parks, Treena McDonald, Andrew Roth, Grace Shen-Tu, Jennifer Vena, Rachel A. Murphy, Parveen Bhatti

**Affiliations:** 1 School of Population and Public Health, University of British Columbia, Vancouver, BC V6T 1Z3 Canada; 2 Population Health Sciences, BC Cancer Research Institute, Vancouver, BC V5Z 1L3 Canada; 3 Pathology and Laboratory Medicine, Faculty of Medicine, University of British Columbia, Vancouver, V6T 1Z7 Canada; 4 Computer Science, Faculty of Science, University of British Columbia, Vancouver, V6T 1Z4 Canada; 5 Basic and Translational Research, BC Cancer Research Institute, Vancouver, BC V5Z 1L3 Canada; 6 Alberta’s Tomorrow Project, Cancer Care Alberta, Alberta Health Services, Calgary, AB T2N 5G2 Canada

**Keywords:** Breast cancer, Biomarkers, Metabolomics, Mass-spectometry, Risk prediction

## Abstract

**Background:**

Metabolomics offers a promising approach to identify biomarkers for timely intervention and enhanced screening of individuals at increased risk of developing breast cancer.

**Methods:**

We conducted a study of 593 female breast cancer cases and 593 matched controls nested in two prospective cohort studies. Mass spectrometry, without liquid chromatography, was used to conduct untargeted metabolomics profiling of serum samples collected, on average, 5.3 years before cancer diagnosis. Logistic regression was used to estimate odds ratios (OR) for a one standard deviation increase of metabolite intensities. Partial least squares discriminant analyses were applied to those metabolites significantly associated with breast cancer to develop risk prediction models.

**Results:**

Associations were evaluated with a total of 837 metabolites. Twenty-four metabolites were significantly associated with overall breast cancer risk, including 13 associated with decreased risk and 11 associated with increased risk. Putative identities of the metabolites included various amino acids (*n* = 3), dietary factors (*n* = 10), fatty acids (*n* = 2), phosplipids (*n* = 4), sex hormone derivatives (*n* = 2), and xenobiotics (*n* = 3). For example, a metabolite identified as acetyl tributyl citrate, a plasticizer in food wrappings, was associated with an increased risk of breast cancer (OR = 1.21; 95% CI: 1.07–1.37). Risk prediction models for overall breast cancer and the various subtypes were found to have modest levels of prediction accuracy (area under the curve ranged from 0.60 to 0.63).

**Conclusions:**

Additional studies are needed to confirm the identities of the metabolites and validate their associations with breast cancer risk. Metabolomics should be evaluated in conjunction with other ‘omics’ technologies for creation of clinically useful risk prediction models.

**Supplementary Information:**

The online version contains supplementary material available at 10.1186/s13058-025-02102-w.

## Introduction

Tools to predict the future occurrence of breast cancer could significantly reduce the burden of disease by supporting targeted prevention strategies and/or enhanced screening for early detection. Current breast cancer risk prediction models based on known breast cancer risk factors (e.g., age, reproductive events, family history, history of breast diseases) have insufficient discriminatory accuracy to support targeted prevention and screening efforts [1]. Metabolomics, which involves the analysis of low-molecular-weight molecules in biological samples, is a promising approach for predicting breast cancer risk since the metabolome can serve as a functional readout of the impact of genetic and environmental factors on mechanisms that contribute to the initiation and progression of breast cancer [2, 3].

Multiple studies of metabolomics, measured in pre-diagnostic blood samples, and breast cancer risk have been recently published [4–13]. For example, in one of the largest studies conducted to date, including 1695 incident breast cancer cases and a subcohort of 1983 women drawn from the Cancer Prevention Study 3, associations with breast cancer were evaluated with 868 metabolites [4]. Compelling associations with various sex hormone derivatives, phospholipids, amino acids, dietary factors, and xenobiotics (e.g., environmental pollutants) were observed. Associations with these general categories of metabolites have been observed across multiple studies, though the specific metabolites identified in the studies have tended to differ. The few replicated metabolites across studies may be attributable, at least in part, to multiple studies using targeted assay platforms that assessed relatively few metabolites or using nuclear magnetic resonance (NMR) platforms which are not as sensitive as mass spectrometry (MS) platforms [14]. Only three studies, two of which used NMR platforms, reported breast cancer risk prediction results [6, 9, 12]. Two of the studies demonstrated high prediction accuracy, one of which was nested within a registry of women with a family history of breast and/or ovarian cancer [9, 12].

By pooling data from two prospective Canadian cohort studies, our primary objective was to identify metabolites, measured in pre-diagnostic blood samples using a high throughput untargeted MS-platform, that were associated with breast cancer risk, including risk of major breast cancer subtypes. We also sought to evaluate the utility of the identified metabolites for breast cancer risk prediction.

## Methods

### Study population

Data for the study came from two prospective longitudinal population cohort studies, the BC Generations Project (BCGP) and Alberta’s Tomorrow Project (ATP). BCGP consists of 29,788 participants from across British Columbia, aged 35 to 69 at time of recruitment, which occurred between 2009 and 2016 [15]. ATP consists of 54,922 participants from across Alberta, aged 35 to 69 at time of recruitment, which occurred in two phases (Phase I, 2000–2008; Phase II, 2009–2015) [16]. During Phase II, newly recruited participants and a portion of previously recruited participants (*n* = 24,075) completed questionnaires similar to those completed by BCGP participants at time of recruitment. These questionnaires collected health and reproductive history, social demographic characteristics, and behavioral factors. As both BCGP and ATP belong to the Canadian Partnership for Tomorrow’s Health (CanPath) [17], these questionnaires underwent an extensive harmonization process. Participants also provided height and weight measurements either via in-person assessments at a study center or through self-report. Participants provided blood samples, which were divided into aliquots of plasma, serum, buffy coat, and red blood cells and stored at -80 °C.

### Study design

**Fig. 1:**
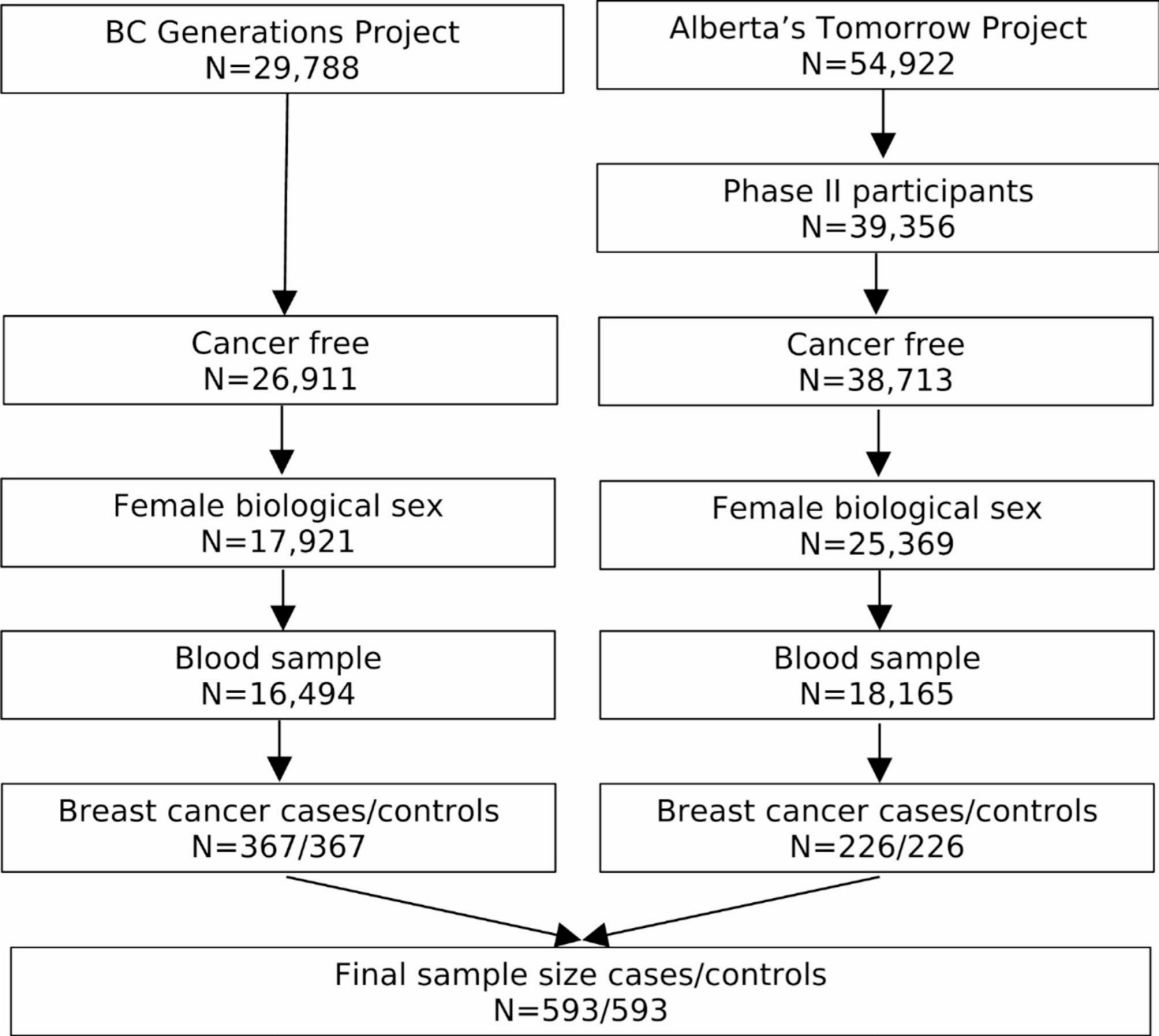
Inclusion criteria for study of serum metabolome and breast cancer risk

Participants were eligible to be included in the current study if they were female, completed a questionnaire (baseline questionnaire for BCGP; Phase II questionnaire for ATP), provided blood samples at time of questionnaire completion, and were cancer-free, except for nonmelanoma skin cancer, at time of questionnaire completion (Fig. 1). A total of 16,494 BCGP and 18,165 ATP participants met these eligibility criteria. Among these participants, cases were selected if they were diagnosed with incident invasive breast cancer. Incident invasive breast cancer cases were identified through annual linkage with each province’s respective Cancer Registry (International Classification of Diseases for Oncology, Third Edition site code: C50.x; morphology codes: 80103, 80323, 80413, 81403, 82003, 82113, 82553, 84013, 84803, 85003, 85033, 85043, 85073, 85203, 85223, 85233, 85243, 85403, 84413, 85753, 89833). Based on morphology codes, cases were classified into four histological subgroups: invasive ductal (85003, 85043, 85073), lobular (85203), mixed ductal/lobular (8522, 8523, 8524, 8255), and other carcinomas. In addition, cases with known hormone receptor expression status were categorized as: luminal A [estrogen receptor positive (ER+) and/or progesterone receptor positive (PR+) and human epidermal growth factor receptor 2 negative (HER2-)], HER2+, and triple-negative breast cancers.

Each case was matched to a single control who was cancer free at the time of case diagnosis, except for nonmelanoma skin cancer, on source cohort, baseline menopause status, age at blood collection (± 2 years), and year of blood collection. Those with missing menopause status (*n* = 16) were classified as premenopausal if age at time of questionnaire completion was under 51 and postmenopausal if age at time of questionnaire completion was 51 – the median age at natural menopause reported in Canada – or older [18].

### Health, lifestyle, and reproductive data

Body mass index (BMI), measured in kg/m^2^, was calculated based on height and weight data collected during in-person assessments (80% of eligible participants). For those without in-person height and weight data, self-reported measures from the harmonized questionnaire were utilized. Other factors obtained from the questionnaire included age at time of questionnaire completion (same as age at blood collection), ethnicity, annual household income, highest attained education, family history of breast cancer (first degree relatives), age at menarche, number of live births, age at first pregnancy, history of oral contraceptive use, history of hormone replacement therapy, alcohol consumption (average over last year), and smoking status. Missing covariate data were assumed to be missing at random and, as such, were imputed using the Multiple Imputation by Chained Equations (MICE) algorithm [19]. All risk factors and the outcome were used as auxiliary variables for imputation. Ten imputed datasets were generated, each with thirty iterations [20].

### Metabolomics data

Non-fasting serum samples (30 µl each) were plated on 96-well plates and shipped to General Metabolics LLC (Boston, Massachusetts). For a randomly selected 5% of participants, an additional sample was included as a blind duplicate for quality control assessment. Metabolites were measured using previously described MS methods [21]. Briefly, each sample was diluted with 180 µl of 80% ethanol in water. 5 µl of the resulting solution was injected twice, consecutively, at a flow rate of 0.150 ml/minute, into an Agilent 6550 iFunnel Quadrupole time-of-flight (Q-TOF) mass spectrometer. Blanks, pooled study samples, and National Institute of Standards and Technology standard reference materials were injected after every 48 study samples for quality assurance. Metabolites were detected as peaks with specific mass-to-charge ratios (m/z), and the intensity of each peak was a measure of the relative abundance of each metabolite. All peaks with m/z between 50 and 1,000 were recorded for each sample. For those samples with missing values for a particular metabolite (i.e., no peak detected in the sample), the missing values were backfilled using the measured intensity in the sample at the recorded m/z for that metabolite.

Mass spectra data were processed by General Metabolics using MATLAB (The Mathworks, Natick, MA, USA). Metabolite annotations were identified by comparing measured m/z to theoretical m/z within 0.001 Da recorded in the Human Metabolome Database (HMDB) and the Kyoto Encyclopedia of Genes and Genomes (KEGG) database and cross-referenced against the Chemical Entities of Biological Interest (ChEBI) database [22–24]. Since no chromatographic separation was performed in tandem with mass spectrometry, it was not possible to distinguish between metabolites with identical molecular formulas.

Metabolites that were missing in over 50% of the study samples were excluded. Coefficients of variation (CVs) were calculated to assess reproducibility based on the blind duplicate samples. For each pair of duplicate samples, the CV of each metabolite was computed by dividing the standard deviation (SD) by the mean ion intensity, and median CVs across all samples were calculated. Metabolites with median CVs above 20% were excluded. Metabolite intensity measures were normalized using a cubic spline method [25].

After normalization, duplicate intensity measures for each metabolite were averaged to obtain a single measurement per participant. To account for the matched study design, the mean intensity measure of each metabolite in a matched pair was set to zero [26]. In addition, each metabolite intensity was divided by the standard deviation across all samples to assess breast cancer risk associated with one standard deviation increase in metabolite intensity.

### Statistical analysis

Associations of each metabolite with breast cancer risk were estimated using unconditional logistic regression adjusted for matching factors [source cohort (BCGP or ATP), menopause status (pre- or post-), age at blood collection (continuous), year of blood collection (2009–2019, 2011–2012, or ≥ 2013)] (R version 4.3.2). Multiple testing was accounted for by applying the Benjamini-Hochberg procedure with the false discovery rate (FDR, also known as q-value) set at 0.1. For metabolites that were deemed significant after multiple testing correction, we fitted separate logistic regression models to investigate the potential for confounding effects by established breast cancer risk factors, including ethnicity (White or Non-white), annual household income (<$50,000, $50,000-<$100,000, or ≥$100,000), highest attained education (high school or less, some post-secondary, or Bachelor’s or higher), family history of breast cancer (yes or no), age at menarche (< 12 years, 12–13 years, or ≥ 14 years), number of live births (continuous), age at first pregnancy (never pregnant, < 25 years, 25–29 years, or ≥ 30 years), oral contraceptive use (ever or never), hormone replacement therapy (ever or never), BMI (< 18.5–24.9 kg/m^2^, 25.0–29.9 kg/m^2^, or ≥ 30 kg/m^2^), alcohol consumption (none, 1–3 times/month, 1–3 times/week, or ≥ 4 times/week), and smoking status (never, past, or current). Analyses were completed on each of the imputed data sets and the resulting odds ratio (OR) estimates were averaged. A pooled Wald-based 95% CI was generated using Rubin’s Rule [27]. Statistically significant metabolites from the overall analysis were also evaluated in the following subgroups: participants who were postmenopausal at time of blood sample collection, cases diagnosed with invasive ductal carcinomas along with their matched controls, and cases diagnosed with luminal A breast cancers and their matched controls. Numbers of other breast cancer subtypes were insufficient to conduct meaningful analyses.

Risk prediction models were developed using partial least squares discrimination analysis (PSL-DA) (R version 4.3.2) [28]. Prediction models were generated using the metabolites found to be statistically significantly associated with breast cancer in the overall logistic regression analysis, with and without established breast cancer risk factors (all models included matching factors). For each model, 80% of the data were used as a training set and 20% were used as a testing set. Pair identifiers were used to split the data to preserve the matched design. For cross-validation, training data were randomly split into 10 equal-sized subsets, each also maintaining case-control matching. Nine subsets were used to train the algorithms across a grid of hyperparameters, and the tenth subset was used to test performance. This process was repeated 10 times, and the hyperparameters that resulted in the highest cross-validated area under the curve (AUC) were used to predict outcome in the testing data set. One-hundred models were generated from 100 independent training-testing partitions. The empirical mean and 95% CI for predictive performance were calculated.

## Results

A total of 593 matched cases and controls – consisting of 226 cases and controls from ATP and 367 cases and controls from BCGP – were included in the study. Distributions of variables for cases and controls, before imputation, are summarized in Table 1. The distributions of breast cancer risk factors were generally similar between cases and controls, though more controls (79.4%) than cases (73.5%) reported no family history of breast cancer. For cases, cancer diagnosis occurred within a mean (SD) of 5.3 (2.6) years after questionnaire completion/blood sample collection, with a mean (SD) age at diagnosis of 62.5 (7.9) years. Hormone receptor status data were missing for 206 (34.7%) breast cancer cases.

**Table 1:** Characteristics of cases and controls

Variable	Case^a^	Control^a^
	*n* = 593	*n* = 593
Cohort, n (%)		
BCGP	367 (61.9%)	367 (61.9%)
ATP	226 (38.1%)	226 (38.1%)
Menopause stats, n(%)		
Premenopausal	167 (28.2%)	167 (28.2%)
Postmenopausal	426 (71.8%)	426 (71.8%)
Age at blood collection, mean (SD)	58.1 (7.9)	58.0 (8.0)
Year of blood collection, n (%)		
2009–2010	150 (25.3%)	148 (25.0%)
2011–2012	190 (32.0%)	201 (33.9%)
≥2013	253 (42.7%)	244 (41.1%)
Ethnicity, n (%)		
White	481 (81.1%)	479 (80.8%)
Non-white	36 (6.1%)	37 (6.2%)
Missing	76 (12.8%)	77 (13.0%)
Annual household income, n (%)		
<$50,000	135 (22.8%)	131 (22.1%)
$50,000-<$100,000	214 (36.1%)	217 (36.6%)
≥$100,000	195 (32.9%)	210 (35.4%)
Missing	49 (8.3%)	35 (5.9%)
Highest attained education, n (%)		
High school or less	> 100 (> 18.6%)	> 100 (> 18.7%)
Some post-secondary	229 (38.6%)	239 (36.6%)
Bachelor’s or higher	218 (32.9%)	214 (35.4%)
Missing	< 10 (< 1.7%)	< 10 (< 1.7%)
Family history of breast cancer, n (%)		
Yes	> 100 (> 18.6%)	108 (18.2%)
No	436 (73.5%)	471 (79.4%)
Missing	< 10 (< 1.7%)	14 (2.4%)
Age at menarche, n (%)		
<12 years	99 (16.7%)	93 (15.7%)
12–13 years	341 (57.5%)	330 (55.6%)
≥14 years	131 (22.1%)	150 (25.3%)
Missing	22 (3.7%)	20 (3.4%)
Number of live births, mean (SD)	1.7 (1.2)	1.9 (1.2)
Missing, n	< 10	< 10
Age at first pregnancy, n (%)		
Never pregnant	96 (16.3%)	90 (15.3%)
<25 years	248 (41.8%)	281 (47.4%)
25–29 years	142 (24.1%)	132 (22.4%)
≥30 years	103 (17.5%)	87 (14.7%)
Oral contraceptive use, n (%)		
Ever	539 (90.9%)	520 (87.7%)
Never	> 50 (< 8.4%)	> 50 (< 8.4%)
Missing	< 10 (< 1.7%)	< 10 (< 1.7%)
Hormone replacement therapy use, n (%)		
Ever	232 (39.5%)	220 (37.2%)
Never	356 (60.5%)	372 (62.5%)
Body mass index, n (%)		
<18.5–24.9 kg/m^2^	257 (43.3%)	271 (45.7%)
25.0–29.9 kg/m^2^	175 (29.5%)	163 (27.5%)
≥30.0 kg/m^2^	115 (19.4%)	117 (19.7%)
Missing	46 (7.8%)	42 (7.1%)
Alcohol consumption frequency/week, n (%)		
None	> 50 (> 8.4%)	> 50 (> 8.4%)
1–3 times/month	208 (35.1%)	197 (33.2%)
1–3 times/week	177 (29.8%)	180 (30.4%)
≥4 times/week	148 (25.0%)	144 (24.3%)
Missing	< 10 (< 1.7%)	< 10 (1.7%)
Smoking status, n (%)		
Never smoker	315 (53.1%)	311 (52.4%)
Past smoker	237 (40.0%)	237 (40.0%)
Current smoker	28 (4.7%)	28 (4.7%)
Missing	13 (2.2%)	17 (2.9%)
Age at breast cancer diagnosis, mean (SD)	62.5 (7.9)	
Time to diagnosis (years), mean (SD)	5.3 (2.6)	
Histological subtype, n (%)		
Invasive ductal	442 (74.5%)	
Lobular	73 (12.3%)	
Mixed ductal/lobular	45 (7.6%)	
Other	33 (5.6%)	
Molecular subtype, n (%)		
ER + and/or PR+, HER2- (Luminal A)	300 (50.6%)	
HER2+	48 (8.1%)	
Triple-negative	39 (6.6%)	
Missing	206 (34.7%)	

Any cells in the table with less than 10 observations were labelled as ‘<10’. Adjacent cells were labelled as ‘>50’ or ‘>100’ to help obscure the low number of observations

A total of 1,676 metabolites were annotated, of which 854 (50.9%) were detected among at least 50% of study samples. Median CVs among duplicate samples ranged from 1.1 to 31.9%, with 17 metabolites (2.0%) having CVs greater than 20%. These metabolites were excluded, leaving 837 metabolites for analysis.

**Fig. 2:**
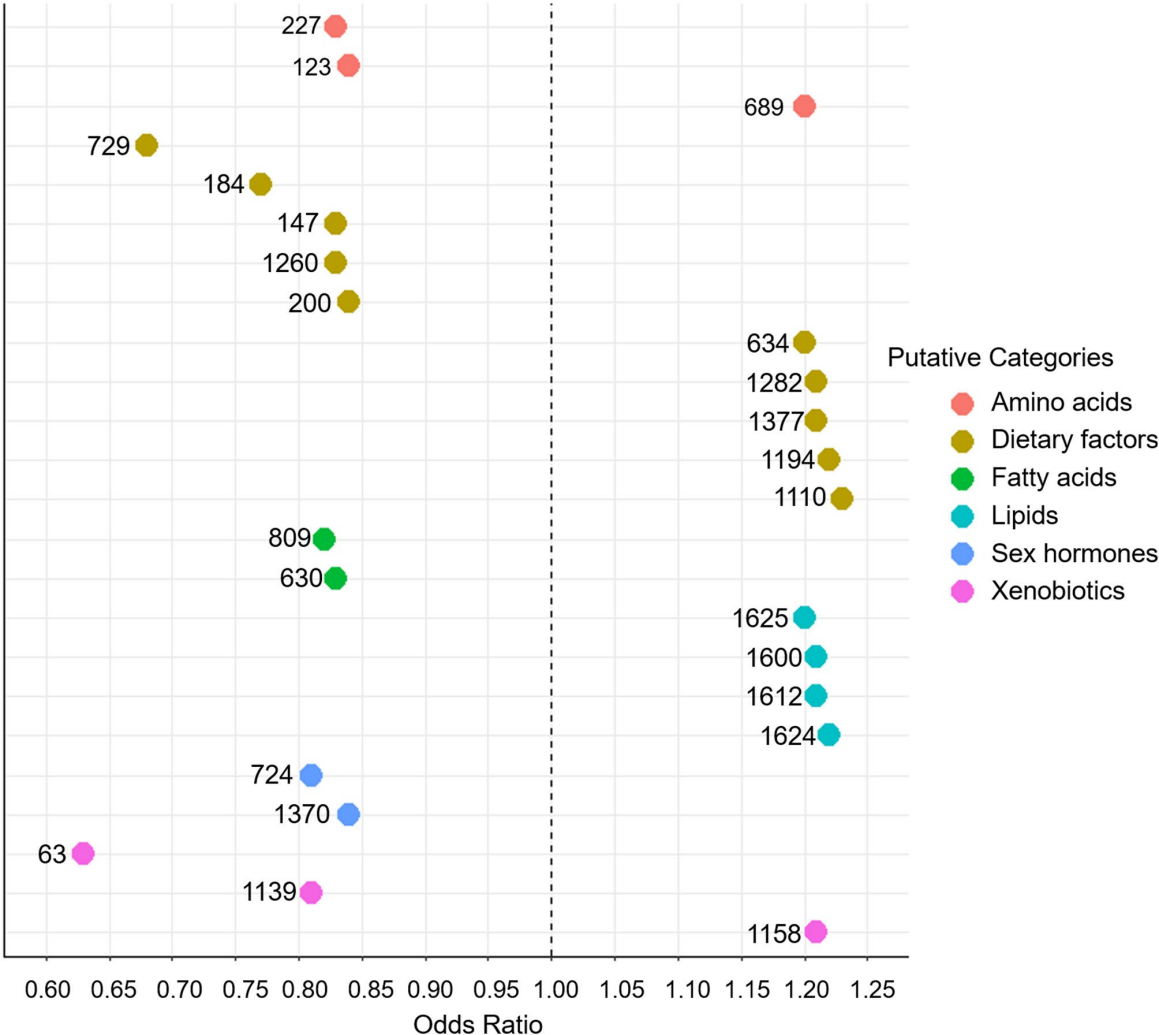
Statistically significant metabolite associations with overall breast cancer risk, grouped by metabolite category. Numbers correspond to metabolite identification numbers (Table 2). Categories are based on the possible identities of metabolites

Associations between each of the 837 metabolites and overall breast cancer risk are presented in Supplementary Table 1. Each metabolite was assigned an identification number, and for each metabolite identification number, Supplementary Table 2 lists potential annotations. The potential annotations were not always distinct metabolites; some were alternative names for the same metabolite. After correcting for multiple comparisons, 24 metabolites were statistically significantly associated with overall breast cancer risk (Table 2). Thirteen metabolites were associated with reduced breast cancer risk, with ORs ranging from 0.63 to 0.84. Metabolite 63 [monomethyl sulfate was only identified annotation (Supplementary Table 2)] had the strongest inverse association (OR = 0.63; 95% CI: 0.51–0.78). Eleven metabolites were positively associated with breast cancer risk, with ORs ranging from 1.20 to 1.23. Metabolite 1110, annotated as multiple glycoside compounds, had the strongest positive association (OR = 1.23 95% CI: 1.09–1.39). No confounders of these associations were identified (addition of covariates listed in Table 1 did not materially impact OR estimates) so final models included only matching factors. Figure 2 displays the ORs for each of the 24 metabolites, grouped by metabolite category. Categories are based on the possible identities of the metabolites (see Discussion for details).

**Table 2:** Associations of significant metabolites with overall breast cancer and breast cancer subgroups

Metabolite ID number^a^		Overall^b^			Postmenopausal^b^				Invasive ductal^b^				Luminal A^b^		
	OR	95% CI	q		OR	95% CI	q		OR	95% CI	q		OR	95% CI	q
63	**0.63**	**0.51**,** 0.78**	**0.01**		**0.57**	**0.44**,** 0.76**	**0.02**		**0.65**	**0.51**,** 0.84**	**0.05**		0.82	0.60, 1.14	0.52
729	**0.68**	**0.57**,** 0.81**	**0.01**		**0.61**	**0.49**,** 0.77**	**0.01**		**0.65**	**0.52**,** 0.82**	**0.04**		0.73	0.59, 0.90	0.12
184	**0.77**	**0.64**,** 0.91**	**0.10**		**0.68**	**0.55**,** 0.84**	**0.04**		0.84	0.70, 1.00	0.34		**0.65**	**0.51**,** 0.85**	**0.09**
724	**0.81**	**0.72**,** 0.92**	**0.10**		**0.75**	**0.64**,** 0.88**	**0.04**		**0.79**	**0.68**,** 0.91**	**0.05**		0.86	0.70, 1.04	0.40
1139	**0.81**	**0.72**,** 0.91**	**0.10**		**0.80**	**0.70**,** 0.93**	**0.10**		0.84	0.74, 0.97	0.20		**0.72**	**0.60**,** 0.87**	**0.06**
809	**0.82**	**0.73**,** 0.92**	**0.10**		**0.77**	**0.67**,** 0.89**	**0.04**		0.82	0.71, 0.94	0.12		0.89	0.76, 1.04	0.44
147	**0.83**	**0.74**,** 0.93**	**0.10**		0.81	0.70, 0.94	0.12		**0.81**	**0.71**,** 0.93**	**0.10**		0.94	0.79, 1.12	0.76
227	**0.83**	**0.74**,** 0.93**	**0.10**		**0.77**	**0.67**,** 0.89**	**0.04**		0.84	0.73, 0.96	0.16		0.82	0.69, 0.97	0.21
630	**0.83**	**0.74**,** 0.94**	**0.10**		0.83	0.72. 0.95	0.13		**0.81**	**0.71**,** 0.93**	**0.10**		0.84	0.72, 0.99	0.25
1260	**0.83**	**0.74**,** 0.93**	**0.10**		0.84	0.73, 0.97	0.18		0.87	0.76, 0.99	0.31		0.81	0.68, 0.96	0.20
123	**0.84**	**0.75**,** 0.94**	**0.10**		**0.80**	**0.69**,** 0.93**	**0.10**		0.86	0.75, 0.98	0.23		0.90	0.76, 1.07	0.52
200	**0.84**	**0.75**,** 0.94**	**0.10**		0.84	0.73, 0.96	0.17		**0.78**	**0.68**,** 0.90**	**0.04**		0.85	0.71, 1.02	0.34
1370	**0.84**	**0.75**,** 0.94**	**0.10**		0.90	0.78, 1.03	0.4		0.89	0.78, 1.01	0.39		0.79	0.67, 0.93	0.14
634	**1.20**	**1.07**,** 1.35**	**0.10**		1.21	1.05, 1.40	0.14		1.18	1.03, 1.35	0.21		**1.32**	**1.11**,** 1.57**	**0.10**
689	**1.20**	**1.07**,** 1.34**	**0.10**		**1.23**	**1.07**,** 1.41**	**0.10**		**1.26**	**1.10**,** 1.43**	**0.05**		1.18	1.02, 1.38	0.24
1625	**1.20**	**1.07**,** 1.34**	**0.10**		1.12	0.97, 1.28	0.39		1.20	1.05, 1.36	0.14		1.09	0.92, 1.28	0.61
1158	**1.21**	**1.07**,** 1.37**	**0.10**		1.15	0.99, 1.34	0.31		1.18	1.02, 1.36	0.23		1.13	0.94, 1.36	0.49
1282	**1.21**	**1.08**,** 1.36**	**0.10**		1.16	1.01, 1.32	0.25		**1.35**	**1.17**,** 1.56**	**0.01**		1.09	0.92, 1.29	0.59
1377	**1.21**	**1.08**,** 1.36**	**0.10**		1.08	0.94, 1.24	0.52		**1.29**	**1.12**,** 1.49**	**0.04**		1.03	0.86, 1.23	0.87
1600	**1.21**	**1.08**,** 1.36**	**0.10**		**1.23**	**1.07**,** 1.42**	**0.10**		1.19	1.04, 1.37	0.16		1.23	1.04, 1.46	0.20
1612	**1.21**	**1.08**,** 1.36**	**0.10**		1.12	0.97, 1.28	0.38		**1.33**	**1.16**,** 1.52**	**0.02**		1.09	0.93, 1.29	0.58
1194	**1.22**	**1.08**,** 1.38**	**0.10**		1.19	1.03, 1.38	0.2		1.19	1.03, 1.37	0.22		1.15	0.96, 1.37	0.43
1624	**1.22**	**1.09**,** 1.37**	**0.10**		1.13	0.99, 1.30	0.33		**1.34**	**1.17**,** 1.54**	**0.01**		1.11	0.95, 1.31	0.50
1110	**1.23**	**1.09**,** 1.39**	**0.10**		1.19	1.02, 1.39	0.22		1.23	1.06, 1.43	0.13		1.09	0.91, 1.31	0.64

^a^See Supplementary Table 2 for potential annotations

^b^Adjusted for source cohort (BCGP or ATP), menopause status (pre- or post-), age at blood collection (continuous), and year of blood collection (2009–2019, 2011–2012, or ≥ 2013)

Among postmenopausal participants (426 case-control pairs), statistically significant associations were observed for ten of the 24 metabolites (Table 2). Eight metabolites were associated with decreased risk of breast cancer, and two metabolites were associated with increased risk of breast cancer. As in the overall analysis, the strongest inverse association was observed with metabolite 63 (monomethyl sulfate, OR = 0.57, 95% CI: 0.44–0.76). Significant positive associations were observed with metabolite 689 (n-[4’-hydroxy-(e)-cinnamoyl]-l-aspartic acid was only identified annotation, OR = 1.23; 95% CI: 1.07–1.41) and metabolite 1600 (annotated as multiple phosphatidylethanolamines, OR = 1.23; 95% CI: 1.07–1.42).

Eleven of the 24 metabolites were statistically significantly associated with invasive ductal carcinoma of the breast (442 case-control pairs), including six associated with reduced risk and five associated with increased risk (Table 2). The strongest inverse associations were observed for metabolites 63 (monomethyl sulfate, OR = 0.65; 95% CI: 0.51–0.84) and 729 (lauroyl diethanolamide was only identified annotation, OR = 0.65; 95% CI: 0.52–0.82). The strongest positive association was observed with metabolite 1282 (multiple potential annotations, OR = 1.35; 95% CI: 1.17–1.56).

Three of the 24 metabolites were found to be statistically significantly associated with risk of luminal A breast cancer (300 case-control pairs), including two associated with reduced risk and one associated with increased risk of breast cancer. The strongest protective association was observed for metabolite 184 (multiple potential annotations, OR = 0.65; 95% CI: 0.51–0.85), while the positive association was observed for metabolite 634 (multiple potential annotations, OR = 1.32; 95% CI: 1.11–1.57).

The AUCs for the breast cancer risk prediction models are provided in Table 3. AUCs ranged from 0.60 to 0.63 for overall breast cancer and for each of the subgroups, with and without inclusion of known breast cancer risk factors.

**Table 3:** Area under the curve (AUC) of risk prediction models for overall breast cancer and breast cancer subgroups

Group	Predictors^a^	AUC (95% CI)
Overall BC	Metabolites	0.62 (0.56–0.70)
	Metabolites and risk factors^b^	0.60 (0.52–0.69)
Postmenopausal	Metabolites	0.63 (0.52–0.71)
	Metabolites and risk factors^b^	0.61 (0.58–0.72)
Invasive ductal	Metabolites	0.62 (0.52–0.71)
	Metabolites and risk factors^b^	0.60 (0.56–0.68)
Luminal A	Metabolites	0.62 (0.46–0.73)
	Metabolites and risk factors^b^	0.60 (0.57–0.71)

^a^All models included matching factors (cohort, menopause status, age at blood collection, year of blood collection

^b^Risk factors included ethnicity, annual household income, highest attained education, family history of breast cancer, age at menarche, number of live births, age at first pregnancy, oral contraceptive use, hormone replacement therapy, BMI, alcohol consumption, and smoking status

## Discussion

We observed multiple metabolites to be associated with increased and decreased risks of breast cancer in our study. No notable differences in direction and strength of associations between metabolites and breast cancer subtypes were noted. Risk prediction models, with and without breast cancer risk factors, had only modest levels of predictive performance.

The strongest overall association was a protective effect observed with metabolite 63, which was identified as monomethyl sulfate. This was counter-intuitive since monomethyl sulfate is a xenobiotic with genotoxic properties found in airborne particulate matter and as an impurity in pharmaceutical manufacturing [29, 30]. Another metabolite, 1158, was associated with an increased risk of breast cancer and was identified as acetyl tributyl citrate. This is a common plasticizer in food wrappings, children’s toys, and medical coatings [31] that has been previously associated with increased breast cancer risk [5]. Metabolite 1139 was associated with a decreased risk of breast cancer, and the only annotation identified was N-benzoyl-D-arginine-4-nitroanilide. Limited information on sources and toxicity of this compound was found, though it was previously identified in a human blood exposome database and is likely of exogenous origin [32].

Multiple metabolites with potential links to dietary intake were associated with breast cancer risk in our study. Metabolite 729, identified as lauroyl diethanolamide, is a derivative of lauric acid, which is commonly found in coconut and palm kernel oils. Consistent with our observation, a previous study linked increased breast cancer risk with lower plasma lauric acid concentrations [33]. We observed metabolites 147 and 200 to be associated with decreased risks of breast cancer. One of the annotations for metabolite 147 was ethyl maltol, a commonly used flavoring in food and e-cigarettes with possible cytotoxic properties [34]. No information on anti-carcinogenic effects of the other annotations for this metabolite was identified. Annotations for metabolite 200 included multiple compounds that, according to the Human Metabolome Database, are naturally occurring in food (e.g. 3-ethyl-5-methoxyphenol) or added to food as flavoring (e.g., 3-(5-methyl-2-furanyl)butanal) [35]. Limited information on the toxicity of these compounds was identified. The potential annotations for metabolite 184, which was associated with a decreased risk of breast cancer, included cinnamate. Cinnamate is a plant compound with multiple anticarcinogenic properties, including suppression of cell proliferation and metastasis [36]. Similarly, one of the annotations for metabolite 1260, which was also associated with a decreased risk of breast cancer, was the plant compound kurarinone, which has anti-inflammatory and anti-oxidative properties [37]. Metabolite 634 was associated with an increased risk of breast cancer; however, all potential annotations were compounds that have been associated with anticarcinogenic effects. For example, one of the annotations was celerin, a coumarin in celery, and coumarins have anti-tumour effects such as inducing apoptosis and inhibition of tumor angiogenesis [38].

Metabolites 1377 and 1194, which were both associated with increased risks of breast cancer, were annotated as Fusicoccin H and atractyloside G, respectively. Both are glycosides produced by fungi and plants; exposure to glycosides would occur primarily through our diet. While atractylosides have anti-cancer properties [39], Fusicoccins have been shown to stabilize protein-protein interactions that may promote cancer cell proliferation and migration [40]. Metabolite 1110, which was also associated with an increased risk of breast cancer, was annotated as multiple glycosides. No information on the carcinogenic properties of these compounds was identified.

Metabolite 1282 was associated with an increased risk of breast cancer. One of its possible annotations was 1a,24R,25-trihydroxyvitamin D2, a metabolite of vitamin D2; vitamin D2 is obtained through our diet. While no information about the carcinogenic impacts of 1a,24R,25-trihydroxyvitamin was found, other vitamin D2 metabolites, such as 1,24(S)-dihydroxyvitamin D2, have been observed to have antitumor activity in animal breast cancer cells [41]. No information suggesting potential carcinogenic effects of the other possible annotations for this metabolite was found.

Metabolites 123 and 689 were identified as oxalurate and n-[4’-hydroxy-(e)-cinnamoyl]-l-aspartic acid, respectively. Oxalurate, which was associated with a decreased risk of breast cancer, is an amino acid while n-[4’-hydroxy-(e)-cinnamoyl]-l-aspartic acid, which was associated with an increased risk of breast cancer, is a derivative of the amino acid aspartate. No previous studies of oxalurate and cancer were identified; however, aspartate has been previously associated with increased risk of breast cancer which is consistent with our finding [42]. Metabolite 227 was associated with a decreased risk of breast cancer. Annotations for the metabolite included various breakdown products of the amino acid tyrosine, such as succinylacetone, which has been shown to have anti-proliferative effects [43].

Metabolites 1600, 1612, 1624, and 1625 were all associated with increased breast cancer risks, and each one was annotated as multiple phosphatidylethanolamines. While previous studies have identified various phospholipids – including phosphatidylethanolamines – to be associated with breast cancer risk, unlike our findings, mostly inverse relationships between these lipids and breast cancer risk have been observed [4, 7, 8].

Metabolite 630 was associated with a decreased risk of breast cancer. One of its possible annotations was 10-hydroxymyristic acid methyl ester, a fatty alcohol of myristic acid which is abundant in milk fat [44]. Contrary to our finding, circulating plasma myristic acid levels were observed to be associated with increased postmenopausal breast cancer risk in a previous study [45]. No information on potential anti-cancer properties of the other possible annotations of this metabolite was identified. One of the annotations for metabolite 809 was arachidonate, an omega-6 polyunsaturated fatty acid. Arachidonate induces anti-inflammatory lipoxins which may suppress proliferation of blood vessels that support tumor growth [46]. Lower blood arachidonate levels have been previously associated with increased postmenopausal breast cancer risk [47]. This is consistent with findings from our study in which metabolite 809 was observed to be associated with a decreased risk of breast cancer among postmenopausal women.

Metabolite 724 was associated with a decreased risk of breast cancer and was annotated as various estrogen metabolites. One of the potential annotations was 2-hydroxyestrone, which is protective against breast cancer since it does not stimulate cell division and, by binding with estrogen receptors, can prevent the proliferative effect of other estrogen metabolites [48]. Metabolite 1370 was also associated with a decreased risk of breast cancer. One of its potential annotations was 11-oxo-androsterone glucuronide, an androgen metabolite [49]. 11-oxygenated androgens have been previously associated with increased risk of breast cancer [50]. No information on anti-carcinogenic effects of the other annotations for this metabolite were identified.

In a previous study of 419 cases and 419 controls, Bro et al. (2015) applied PLS-DA to NMR and breast cancer risk factor data to create risk prediction models [9]. In that study, a total of 129 spectral regions were identified from the NMR data and included as separate variables in the analysis (the number of metabolites represented by these data was not indicated). The resulting model had good prediction accuracy for overall breast cancer (AUC = 0.89); models for breast cancer subtypes were not generated.

In a study of 791 breast cancer cases and 791 controls, Jobard et al. (2021) also applied PLS-DA to NMR data [6]. NMR spectra were reduced to 8500 bins, each included as a separate variable in the analysis (43 metabolites were identified from the NMR data). The overall model and model focused on postmenopausal cases had poor prediction accuracies (AUC = 0.51 and 0.53, respectively), while a model focused on premenopausal cases had modest prediction accuracy (AUC = 0.61). We did not have enough premenopausal participants in our study to support meaningful analyses. The lower predictive performance reported by Jobard et al. (2021) and our study compared to Bro et al. (2015) may stem from differences in the time interval between blood collection and cancer diagnosis. Shorter intervals are more likely to capture cases with overt disease, potentially resulting in greater alterations in metabolomic profile. While two to five years passed between blood collection and cancer diagnosis for cases in Bro et al. (2015), up to 12.75 years passed between blood collection and cancer diagnosis for cases in Jobard et al. (2021). The time intervals in our study are more consistent with those of Jobard et al. (2021) (up to 12 years). Given the limited sample size, we did not conduct effect modification analyses to determine if associations differed by time interval between blood collection and cancer diagnosis in our study.

Wu et al. (2024) conducted a small-scale study of 40 cases and 70 age-matched controls from a registry of women with a family history of breast and/or ovarian cancer. Eight metabolites were found to be associated with breast cancer risk before multiple comparisons adjustment [12]. When adding these metabolites to a PLS-DA model including age and the Breast and Ovarian Analysis of Disease Incidence and Carrier Estimation Algorithm (BOADICEA) risk score, the AUC improved from 0.66 to 0.83. While the study did include cases who were diagnosed with breast cancer more than 10 years after blood sample collection, the higher predictive performance as compared to our study may be because Wu et al. (2024) focused on a high-risk population.

Strengths of our study include availability of detailed harmonized data from two well-characterized cohort studies and use of a highly efficient and cost-effective approach with which to conduct untargeted metabolomics analyses and identify promising markers of cancer risk. Ours is also one of the few studies to have used metabolomics data to develop breast cancer prediction models. Given that previous studies used PLS-DA to develop prediction models [6, 9, 12], we did the same to enhance comparability of results. There are, however, multiple classification methods that can be used to develop prediction models, each with their own strengths and weaknesses [51, 52].

Because the metabolomics assay did not incorporate chromatography, we were unable to specifically identify each of the metabolites for which statistically significant associations were observed. This made it impractical to conduct meaningful pathway analyses. Ultimately, a follow-up study incorporating chromatography will be needed to specifically identify metabolites of interest. Previous studies have shown that non-fasting samples, as used in our study, lead to increased variability in metabolite measures [53, 54], potentially masking associations with disease risk. Sample sizes for the subgroup analyses were limited, particularly when examining the luminal A subtype of breast cancer; unfortunately, we were missing hormone receptor status data for a large number of cases.

We observed multiple biologically plausible associations between metabolites and breast cancer risk. However, prediction models generated using these metabolites were found to have modest discrimination accuracy. Multi-omics approaches that incorporate metabolomics data may better capture the complex transformations associated with cancer development [55] and, may, therefore, be better suited for developing useful risk prediction models.

## Supplementary Information

Below is the link to the electronic supplementary material.

Supplementary Material 1

Supplementary Material 2

## Data Availability

Data utilized in this manuscript are available through data access applications to the BC Generations Project (https://bcgpresearch.ca/) and Alberta’s Tomorrow Project (https://myatpresearch.ca/).

## Abbreviations

OR: Odds ratio
CI: Confidence interval
NMR: Nuclear Magnetic Resonance
MS: Mass spectrometry
BCGP: BC Generations Project
ATP: Alberta’s Tomorrow Project
CanPath: Canadian Partnership for Tomorrow’s Health
ER+: Estrogen receptor positive
PR+: Progesterone receptor positive
HER2-: Human epidermal growth factor receptor 2 negative
HER2+: Human epidermal growth factor receptor 2 positive
BMI: Body mass index
MICE: Multiple imputation by chained equations
Q-TOF: Quadrupole time-of-flight
m/z: Mass-to-charge ratios
HMDB: Human Metabolome Database
KEGG: Kyoto Encyclopedia of Genes and Genomes
ChEBI: Chemical Entities of Biological Interest
CV: Coefficient of variation
FDR: False discovery rate
PSL-DA: Partial least squares discrimination analysis
AUC: Area under the curve
SD: Standard deviation
